# High-normal P_a_CO_2_ values might be associated with worse outcome in patients with subarachnoid hemorrhage – a retrospective cohort study

**DOI:** 10.1186/s12883-020-1603-0

**Published:** 2020-01-20

**Authors:** Tilman Reiff, Oliver Barthel, Silvia Schönenberger, Sibu Mundiyanapurath

**Affiliations:** Department of Neurology, Heidelberg University Hospital, Im Neuenheimer Feld 400, D-69120 Heidelberg, Germany

**Keywords:** Subarachnoid hemorrhage, Hypercapnia, Carbon dioxide reactivity, Risk factors, Outcome, Partial pressure of carbon dioxide, Subarachnoid hemorrhage, hypercapnia, carbon dioxide reactivity, ventilation, outcome, aneurysm.

## Abstract

**Background:**

While both hypercapnia and hypocapnia are harmful in patients with subarachnoid hemorrhage (SAH), it is unknown whether high-normal P_a_CO_2_ values are better than low-normal values. We hypothesized that high-normal P_a_CO_2_ values have more detrimental than beneficial effects on outcome.

**Methods:**

Consecutive patients with aneurysmal subarachnoid hemorrhage (aSAH) requiring mechanical ventilation treated in a tertiary care university hospital were retrospectively analyzed regarding the influence of P_a_CO_2_ on favorable outcome, defined as modified Rankin scale score < 3 at discharge. Primary endpoint was the difference in the proportion of P_a_CO_2_ values above 40 mmHg in relation to all measured P_a_CO_2_ values between patients with favorable and unfavorable outcome.

**Results:**

150 patients were included. Median age was 57 years (p25:50, p75:64), median Hunt-Hess score was 4 (p25:3, p75:5). P_a_CO_2_ values were mainly within normal range (median 39.0, p25:37.5, p75:41.4). Patients with favorable outcome had a lower proportion of high-normal P_a_CO_2_ values above 40 mmHg compared to patients with unfavorable outcome (0.21 (p25:0.13, p75:0.50) vs. 0.4 (p25:0.29, p75:0.59)) resulting in a lower chance for favorable outcome (OR 0.04, 95% CI 0.00–0.55, *p* = 0.017). In multivariable analysis adjusted for Hunt-Hess score, pneumonia and length of stay, elevated P_a_CO_2_ remained an independent predictor of outcome (OR 0.05, 95% CI 0.00–0.81, *p* = 0.035).

**Conclusions:**

A higher proportion of P_a_CO_2_ values above 40 mmHg was an independent predictor of outcome in patients with aSAH in our study. The results need to be confirmed in a prospective trial.

## Background

Subarachnoid hemorrhage (SAH) has a high mortality rate and patients suffering from SAH show high rates of disability [1–5]. Many patients endure further neurologic deterioration in the intensive care unit (ICU) after initial treatment [4]. Therefore, knowledge of relevant prognostic factors during treatment is essential. Differences in ventilation are one of the factors affecting clinical outcome. Both hypercapnia and hypocapnia have been shown to be correlated with worse outcome [6–9]. Hypercapnia causes an increase of intracranial pressure (ICP) by vasodilatation [8, 10–12], even when cerebral autoregulation is deranged as in aneurysmal SAH (aSAH) [13–16]. It can lead to secondary brain injury and worse outcome [6, 17]. Furthermore, other effects of hypercapnia as acidosis with negative effects on brain metabolism contribute to the deleterious effects of hypercapnia [12, 18]. On the other hand, hypercapnia causes dilatation of arterial cerebral vessels with enhanced cerebral blood flow (CBF) putatively improving cerebral oxygenation. A recent retrospective study on patients with SAH discovered that patients with arterial partial pressure of carbon dioxide (P_a_CO_2_) values above 48 mmHg had a lower rate of favorable clinical outcome [19]. It therefore remains doubtful whether the positive effects of hypercapnia through increased CBF leading to increased brain oxygenation which might prevent ischemic lesions outweigh the detrimental effects. Prolonged hypocapnia induced by hyperventilation, on the other hand, has been shown to be harmful for patients with SAH as well [6, 12, 19]. An induced reduction of P_a_CO_2_ below 35 mmHg does not improve clinical outcome in these patients. CBF decreases to levels that can cause ischemia and the reduction of intracranial pressure is not only temporary but can also cause rebound intracranial hypertension when normocapnia is restored. Moreover, hypocapnia has deleterious effects on lung compliance, airway resistance, myocardial oxygen supply and systemic oxygenization [6]. Therefore, a pressure within the normal range seems to be reasonable. Many neurointensivists prefer a low-normal P_a_CO_2_ of 35–40 mmHg based on theoretical assumptions and clinical practice [20]. There is not enough evidence to answer the question whether high-normal (40–45 mmHg) or low-normal P_a_CO_2_ values (30–35 mmHg) are beneficial regarding clinical outcome. Currently, there are no guideline recommendations regarding optimal P_a_CO_2_ values within the normal range in intubated patients with SAH [21, 22]. An experimental study showed that even small changes in P_a_CO_2_ can cause changes in the microcirculation. An increase of the P_a_CO_2_ to a median of 45 mmHg led to a dilatation of capillaries [23]. Consequently, it is plausible that high-normal P_a_CO_2_ values may have a different effect on outcome than low-normal values. Another recent study focused on end-tidal CO_2_ values during the coiling or clipping procedure and found no association with clinical outcome [24]. The results of this study imply that P_a_CO_2_ values might have to be studied for a longer period of time and not only for the time of intervention / surgery.

We hypothesized that detrimental effects of high-normal P_a_CO_2_ values (P_a_CO_2_ > 40 mmHg) outweigh the benefits on clinical outcome in patients with SAH. Therefore, our aim was to study the association between the proportion of P_a_CO_2_ values > 40 mmHg during the entire time of mechanical ventilation and clinical outcome.

## Methods

### Patient selection

Consecutive patients suffering from aSAH who were treated in the neurological intensive care unit in the Department of Neurology of a tertiary care university hospital from 12/2006 to 01/2018 were retrospectively analyzed. All information was retrieved from our hospital database. Patients were included if they suffered from aSAH and had to be mechanically ventilated and sedated. We excluded patients if a palliative care approach was begun immediately after the initial computed tomography (CT), because we assumed that P_a_CO_2_ values and frequency of arterial blood gas sampling would be different in these patients. Patients’ pre-treatment conditions including risk factors, severity of the aSAH, relevant medication, age, sex, familial subarachnoid hemorrhage, alcohol abuse, arterial hypertension, use of acetylsalicylic acid (ASA), anticoagulation, Hunt-Hess score, modified Rankin scale score (mRS) before admission, aneurysm characteristics and aneurysm treatment were recorded. All patients received nimodipine for vasospasm and delayed cerebral ischmia (DCI) prophylaxis.

### Outcome parameters

The primary endpoint analysis was performed comparing the proportion of P_a_CO_2_ values above 40 mmHg in relation to all P_a_CO_2_ values between patients with favorable and unfavorable outcome. The value of 40 mmHg P_a_CO_2_ in blood gas analysis was chosen being midway between the thresholds of the normal range (35–45 mmHg). Favorable outcome was defined as a modified Rankin Scale score at discharge of 0–2, implying that the patient is independent in the activities of daily living. If a patient had a mRS before admission of 3 and was discharged with a mRS of 3, this was also considered to be a favorable outcome (back to baseline). The mRS at discharge was assessed by the treating physicians who were blinded to this analysis but not to clinical data of the patient. All variables in the database were predefined before the analysis.

### Other parameters

Mechanical ventilation in all patients was started in pressure-controlled mode. Parameters were adjusted to guarantee lung-protective ventilation and modified according to the attending neurointensivist. Arterial blood gas analysis was performed regularly in clinical routine, roughly every two hours. 2 ml of blood were drawn into a tube (Sarstedt, Nümbrecht, Germany) and injected into a blood gas system (RAPIDPoint 500®, Siemens Healthineers, Erlangen, Germany). All blood gas analysis values of the patient obtained during mechanical ventilation were included in the analysis. Vasospasms were defined as a flow rate of > 200 cm/s in transcranial ultrasound, performed in routine clinical workup with a 2 MHz pulse-wave probe using the SONARA system (medilab®, Estenfeld, Germany). The frequencies used in the study were collected by probing the middle cerebral artery at 50–55 mm depth or the anterior cerebral artery at 70–75 mm depth. Ultrasound was performed by a neurologist or a technician with extensive experience. A re-bleeding had to be diagnosed using CT by an experienced neuroradiologist. CT-imaging was performed using a 64-row multislice CT (Somatom Definition AS®, Siemens Healthineers, Erlangen, Germany) at 120 kiloVolt in X-care technique (automatically adjusting the tube current to reduce radiation dose). Delayed cerebral ischemia was scored positive if a new cerebral infarction was seen on CT after the initial treatment. Symptoms were not taken into account for the definition of DCI as most of the patients were ventilated and the results might have been distorted with more DCI occurring in patients who can report symptoms. Enlargement of the ventricles on CT, described by an experienced neuroradiologist, was recorded as hydrocephalus. Fever was defined as temperature > 38 °C. A standard operating procedure was used lowering temperatures > 37.2 °C, potentially lowering the number of patients with fever. ICP was measured using pressure domes and Infinity® monitors from Dräger, Lübeck, Germany. The Horowitz index was calculated as the ratio of arterial partial pressure of oxygen (P_a_O_2_) and the fraction of oxygen used during ventilation in the inhaled air (FiO_2_).

### Statistical analysis

Univariable analysis was performed with binary logistic regression and Chi^2^-test depending on the distribution. When cell frequency for categorical variables was < 5, the Fisher exact test was used. Multivariable analysis was performed using binary logistic regression with favorable outcome being the dependent variable. The predictive power and discriminating capability of the regression model was tested with the area under the Receiver Operating Characteristic curve (AUROC) and Hosmer-Lemeshow test. Due to the small sample size, we only included the variable of interest (proportion of P_a_CO_2_ values > 40 mmHg), two main confounding factors (pneumonia and length of stay) and the main predictor of outcome (Hunt-Hess score). Reported *p*-values are two-sided and the alpha-level was defined as 0.05. Analyses were performed with STATA/IC 13.0 (College Station, Texas, US).

**Fig. 1:**
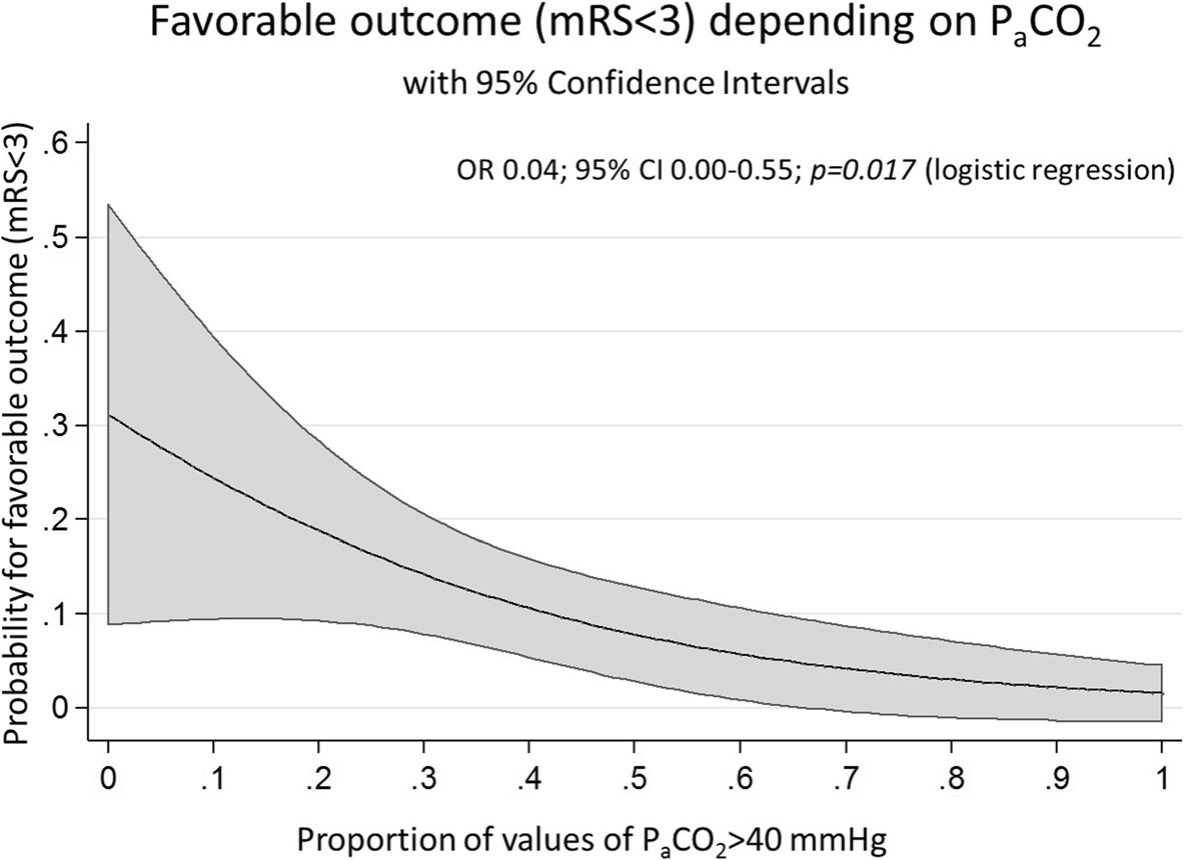
Favorable outcome depending on proportion of values of P_a_CO_2_ > 40 mmHg

The graph of Fig. 1 was prepared as follows: Firstly, logistic regression was calculated in STATA with good outcome as dependent variable and proportion of P_a_CO_2_ values > 40 mmHg as independent variable. Secondly, predictions of odds ratios for good outcome as well as 95% confidence intervals were calculated for every proportion of P_a_CO_2_ values > 40 mmHg between 0 and 1 in 0.01 steps with the “margin” command in STATA. Calculated predictions with confidence intervals were represented graphically by the “marginsplot” command in STATA.

## Results

### Patient characteristics

Of 172 patients with SAH, 22 patients had to be excluded because they did not have aneurysmal SAH. Accordingly, 150 patients were included in the analysis. Median patients’ age was 57 years (p25: 50, p75: 64). 107 (71%) were female. The mRS before admission was 0 in 117 (78%) patients, 1 in 11 (7%) patients, 2 in 5 (3%) patients and 3 in one (1%) patient (median 0, p25: 0, p75: 0). The Hunt-Hess score on admission was 1 in 14 (9%), 2 in 15 (10%), 3 in 44 (29%), 4 in 26 (17%) and 5 in 51 (34%) patients (median 4, p25: 3, p75: 5). 87 (58%) patients suffered from hypertension. Alcohol addiction was recorded in 12 (8%) patients. 5 (3%) patients had first-degree relatives with aSAH. 16 (11%) patients were treated with aspirin and 6 (4%) received oral anticoagulation.

### Aneurysm characteristics

Median aneurysm size was 6 mm (p25: 4, p75: 8.5). Aneurysms causing the SAH were predominantly located in the anterior communicating artery (50, 33%) followed by the middle cerebral artery (29, 19%). More details are presented in Table 1.

**Table 1:** Aneurysm Characteristics; *n* = 150

	median (p25; p75)
Aneurysm size (mm)	6 (4; 8.5)
Aneurysm location	n (%)
Anterior communicating artery	50 (33)
Middle cerebral artery	29 (19)
Posterior communicating artery	18 (12)
Basilar artery	17 (11)
Posterior inferior cerebellar artery	10 (7)
Internal carotid artery	10 (7)
Anterior cerebral artery	6 (4)
Vertebral artery	4 (3)
Posterior cerebral artery	3 (2)
Superior cerebellar artery	2 (1)
Anterior inferior cerebellar artery	1 (1)

Most aneurysms were treated with coiling (55%) followed by clipping (34%). In 11 patients (7%) no treatment for the aneurysm was performed, because of massive rebleeding after placement of external ventricular drains (EVD) or unsuccessful coiling / clipping. Of the 6 patients who were anticoagulated 3 received prothrombin complex concentrate and /or vitamin K. Details can be found in Table 2.

**Table 2:** Aneurysm/SAH treatment; *n* = 150

	n (%)
Coiling	83 (55)
Clipping	51 (34)
Coiling and clipping	4 (3)
Flow diverter	1 (1)
No intervention	11 (7)
Prothrombin complex concentrate	3 (2)
Vitamin K	1 (1)

### Complications and monitoring parameters

Regarding complications during hospitalization, 92 (62%) of the patients suffered from vasospasms, 122 (81%) from hydrocephalus, in 141 (94%) patients received a ventricular or lumbar drain, in 36 patients (24%) rebleeding occured, 74 (49%) got diagnosed with DCI and 88 (59%) suffered from pneumonia.

All patients were ventilated and sedated (mostly propofol/remifentanil or midazolam/sufentanil). Routine neuromuscular block was only used for intubation. Ventilation was mainly performed using pressure control and protective tidal volumes (7.2 ml / kg body weight [p25: 6.2, p75: 8.3]).The majority of patients had P_a_CO_2_ values within the normal range of 35–45 mmHg (median 39 mmHg, p25: 37.45, p75: 41.4). Only 5 (3%) patients were above and 9 (6%) patients were below the normal range. Median partial pressure of arterial oxygen (P_a_O_2_) was 99.45 mmHg (p25: 94.5, p75: 108.0). Median fraction of inspired oxygen (FiO_2_) was 35 (p25: 30, p75: 40). Median ventilation time was 13.4 days (p25: 2.9, p75: 21.2) and median length of stay was 19 days (p25: 12, p75: 26).

Intracranial pressure (ICP) was not different in patients with a median P_a_CO_2_ of > 40 mmHg compared to patients with a P_a_CO_2_ of ≤40 mmHg (8 [p25: 4, p 75: 10] vs. 7 [p 25: 5, p75: 11] mm Hg, respectively, *p* = 0.228). Moreover, pH was also not significantly different between these two groups (7.43 [p25: 7.40, p75: 7.44] vs. 7.43 [p25: 7.40, p75: 7.45] *p* = 0.248). However, a difference was found in base excess (2.35 [p25: 1.4, p75: 3.4] vs. 0.00 [p25: − 2.05, p75: 1.8] *p* < 0.001). Delayed cerebral ischemia (DCI) was not significantly associated with the proportion of P_a_CO_2_ values > 40 mmHg (OR 2.70, 95% CI: 0.58–12.49, *p* = 0.204). Furthermore, there was no difference in P_a_O_2_ and P_a_CO_2_ values between patients with and without DCI (Additional file 1: Table S1). Additional parameters and differences between patients with favorable and unfavorable outcome can be found in Table 3.

**Table 3:** Analysis of factors regarding favorable outcome (mRS < 3) using univariable regression or Fisher’s exact test; *n* = 150

	favorable outcome (*n* = 17) median or *n* (p25; p75 or %)	unfavorable outcome (*n* = 133) median or *n* (p25; p75 or %)	OR (95% CI)	p
*Baseline characteristics*				
Age (median, y)	58 (50; 63)	57 (50; 64)	1.00 *(0.96–1.05)*	*0.884*
Female sex (n)	13 (76%)	94 (71%)	1.35 *(0.41–4.39)*	*0.620*
First degree relatives with SAH (n)	0	5 (4%)		*1.000*^±^
mRS before admission (median)	0 (0; 0)	0 (0; 0)	1.08 *(0.40–2.87)*	*0.883*
Addiction to alcohol (n)	3 (18%)	9 (7%)	2.95 *(0.71–12.20)*	*0.135*
Arterial hypertension (n)	10 (59%)	77 (58%)	1.04 *(0.37–2.90)*	*0.942*
Aspirin (n)	1 (6%)	15 (11%)	0.49 *(0.06–4.00)*	*0.506*
Oral anticoagulation (n)	0	6 (5%)		*1.000*^±^
Aneurysm size (median, mm)	5 (4; 7)	6 (4; 9)	0.87 *(0.73–1.04)*	*0.132*
Hunt-Hess score on admission (median)	3 (2; 3)	4 (3; 5)	0.60 *(0.40–0.88)*	0.010*
Aneurysm location				
Anterior communicating artery	7 (41%)	43 (32%)	2.60 (0.30–22.87)	*0.388*
Middle cerebral artery	2 (12%)	27 (20%)	1.19 (0.10–14.14)	*0.893*
Posterior communicating artery	3 (18%)	15 (11%)	3.20 (0.30–34.24)	*0.336*
Basilar artery	1 (6%)	16 (%)	base	
Posterior inferior cerebellar artery	0	10 (8%)		
Internal carotid artery	1 (6%)	9 (7%)	1.78 (0.10–31.98)	*0.696*
Anterior cerebral artery	0	6 (5%)		
Vertebral artery	1 (6%)	3 (2%)	5.33 (0.26–110.80)	*0.279*
Posterior cerebral artery	0	3 (2%)		
Superior cerebellar artery	1 (6%)	1 (1%)	16.00 (0.52–494.00)	*0.113*
Anterior inferior cerebellar artery	1 (6%)	0		
Treatment modality				
Coiling	12 (71%)	71 (53%)	1.55 *(0.51–4.71)*	*0.435*
Clipping	5 (29%)	46 (35%)	*base*	
Coiling and clipping	0	4 (3%)		
Flow diverter	0	1 (1%)		
No intervention	0	11 (8%)		
*Complications*				
Hydrocephalus (n)	14 (82%)	108 (81%)	1.08 *(0.29–4.05)*	*0.909*
Pneumonia (n)	6 (35%)	82 (62%)	0.34 *(0.12–0.97)*	0.044*
Fever (h, median)	15 (3; 25)	8 (1; 45)	1.00 *(0.99–1.01)*	*0.506*
Vasospasm (n)	11 (65%)	82 (62%)	1.14 *(0.40–3.27)*	*0.807*
Rebleeding (n)	1 (6%)	35 (26%)	0.18 *(0.02–1.37)*	*0.097*
Seizure (n)	2 (12%)	41 (31%)	0.30 *(0.07–1.37)*	*0.120*
Delayed cerebral ischemia (n)	4 (24%)	70 (53%)	0.28 *(0.09–0.89)*	0.032*
Intraparenchymal hemorrhage (n)	2 (12%)	47 (35%)	0.24 *(0.05–1.11)*	*0.068*
Need for ventricular/lumbar drainage (n)	15 (88%)	126 (95%)	0.42 *(0.08–2.19)*	*0.301*
Length of stay (d, median)	21 (16; 30)	19 (11; 25)	1.01 *(0.99–1.04)*	*0.285*
*ventilation parameters*				
Horowitz index (P_a_O_2_/FiO_2_, median)	360 (297; 395)	277 (225; 329)	1.01 *(1.00–1.02)*	0.005*
Proportion of values of P_a_CO_2_ > 40 mmHg	0.21 (0.13; 0.5)	0.4 (0.29; 0.59)	0.04 *(0.00–0.55)*	0.017*
Driving pressure (mbar, median)	13 (12; 14)	13 (12; 15)	0.90 (0.73–1.12)	*0.347*
Tidal volume (ml, median)	550 (499; 592)	520 (473; 579)	1.00 (1.00–1.01)	*0.357*
pH (median)	7.42 (7.39; 7.44)	7.43 (7.40; 7.45)		*0.223*^*◊*∫^
Ventilation time (h, median)	42 (21; 84)	346 (171; 538)	0.99 *(0.99–1.00)*	0.001*
FiO_2_ (median)	0.30 (0.30–0.35)	0.35 (0.30–0.40)	0.90 (0.82–0.99)	0.034*
Intracranial pressure (mm Hg, median)	5 (3; 7)	8 (5; 11)	0.84 (0.73–0.96)	0.013*

*OR* odds ratio and 95% CI; all *p*-values derived from logistic regression except ^±^Fisher’s exact test; *significant; *ASA* Acetylsalicylic acid, ^*◊*^*Mann-Whitney-U test;* ^∫^ univariable logistic regression did not converge

### Outcome

The mRS at discharge was 0 in 1 patient (1%), 1 in 6 (4%), 2 in 9 (6%), 3 in 5 (3%), 4 in 14 (9%), 5 in 65 (43%) and 6 in 50 (33%) patients. 17 patients (11%) had favorable outcome (16 having a mRS < 3 and one patient with back to baseline mRS of 3). In univariable logistic regression the proportion of P_a_CO_2_ values > 40 mmHg was significantly associated with outcome. A higher proportion of values above 40 mmHg lead to a reduced chance for favorable outcome (OR 0.04; 95% CI 0.00–0.55, *p* = 0.017; Fig. 1). Further parameters significantly associated with outcome in univariable analysis were: Hunt-Hess score on admission (OR 0.60; 95% CI 0.40–0.88, *p* = 0.010), pneumonia (OR 0.34; 95% CI 0.12–0.97, *p* = 0.044), delayed cerebral ischemia (OR 0.28; 95% CI 0.09–0.89, *p* = 0.032), ventilation time (OR 0.99; 95% CI 0.99–1.00, *p* = 0.001), Horowitz index (OR 1.01; 95% CI 1.00–1.01, *p* = 0.007), FiO_2_ (OR 0.90, 95% CI 0.82–0.99, *p* = 0.034) and ICP (OR 0.84; 95% CI 0.73–0.96, *p* = 0.013). Location of aneurysm and treatment modality were not significantly associated with outcome. For more details see Table 3.

### Multivariable analysis

In multivariable analysis we adjusted for the main predictors of outcome and major sources of bias for the primary endpoint proportion of P_a_CO_2_ > 40 mmHg (Hunt-Hess score on admission, length of hospital stay and occurrence of pneumonia). A higher proportion of P_a_CO_2_ values > 40 mmHg remained an independent negative predictor for favorable outcome (OR 0.05; 95% CI 0.00–0.81, *p* = 0.035). The Hunt-Hess score on admission and pneumonia were also independent negative predictors for favorable outcome (Table 4). An AUROC of 0.79 and the Hosmer-Lemeshow test revealed a strong predictive capacity of the logistic regression model (*p* = 0.684).

**Table 4:** Multivariable analysis of factors regarding favorable outcome (95% CI; *n* = 150)

	OR (95% confidence interval)	*p*
Proportion of values of P_a_CO_2_ > 40 mmHg	0.05 (0.00–0.81)	0.035*
Hunt-Hess score on admission	0.56 (0.37–0.86)	0.008*
Length of hospital stay (d)	1.02 (0.99–1.06)	*0.204*
Pneumonia	0.26 (0.08–0.88)	0.030*

*OR* odds ratio; all *p*-values logistic regression; *significant

### Sensitivity analysis

As ventilation time was also significantly associated with outcome in univariable analysis, we performed another multivariable analysis using logistic regression with favorable outcome as dependent variable. Due to the collinearity of length of stay and ventilation time we omitted length of stay in this analysis. Furthermore, the proportion of P_a_CO_2_ values already includes a correction for the time of ventilation so that we used the absolute number of P_a_CO_2_ values > 40 mmHg as independent variable instead. The absolute number of P_a_CO_2_ values > 40 mmHg was again an independent negative predictor for favorable outcome (OR (per increase of 10 values) 0.53, 95% CI 0.29–0.99, *p* = 0.048), adjusted for initial Hunt-Hess score, pneumonia and time of ventilation.

## Discussion

In our study population, a higher proportion of high-normal P_a_CO_2_ values (40–45 mmHg) was significantly associated with worse outcome. In multivariable analysis, P_a_CO_2_ remained an independent negative predictor for favorable outcome after adjustment for Hunt-Hess score, length of hospital stay and the occurrence of pneumonia. In our sensitivity analysis, the absolute number of P_a_CO_2_ values > 40 mmHg were again an independent negative predictor of favorable outcome, adjusted for initial Hunt-Hess score, pneumonia and time of ventilation.

So far, there has been substantial research on hypocapnia and hypercapnia in patients with SAH. Some scientists argue that higher P_a_CO_2_ values might increase CBF and decrease the likelihood for cerebral ischemia [25, 26], which would contradict our results. A recent publication showed a benefit of controlled hypercapnia comparing patients with P_a_CO_2_ values of 30 to 40, 50 and 60 mmHg lasting one hour between day 4 and 14 after SAH with elevation of cerebral blood flow and brain tissue oxygen saturation without relevant increase of ICP [25]. However, all patients also had a ventricular drain so that increased ICP might have been prevented by an increased drainage. Additionally, the brain tissue oxygen saturation was measured transcutaneously. This measurement method is imprecise in measuring local effects. One study from our hospital showed that it did not record the effect of thrombectomy in patients suffering from acute ischemic stroke due to large vessel occlusion [27]. Most importantly, clinical outcome was not an outcome of the study and hypercapnia in our study persisted over longer periods of time and is therefore not comparable to brief episodes of hypercapnia with P_a_CO_2_ values returning to baseline within one hour. The detrimental effects of hypercapnia have been demonstrated by a recent study that examined the outcome of patients with aneurysmal SAH and focused on the hyperventilated and hypoventilated patients [19]. 158 patients were retrospectively analyzed. The authors showed that P_a_CO_2_ values higher than 48.3 mmHg and lower than 30.2 mmHg were associated with unfavorable outcome at discharge defined as mRS score of 3–6.

The detrimental effects of elevated P_a_CO_2_ could be explained by a reversed Robin-Hood-phenomenon which has been described in cerebral ischemia [28]. While vessels with vasospasm do not dilate, unaffected vessels dilate leading to shunting of blood away from already oligemic or ischemic areas, worsening the effect of vasospasms. In an experimental study, hypercapnia led to dilatation of small vessels in sham-operated animals. In animals with experimental SAH, however, higher P_a_CO_2_ levels had no effect on vessel diameter [29]. In our study we could not show a significant association of the proportion of P_a_CO_2_ values > 40 mmHg with DCI. Moreover, the median P_a_O_2_ and P_a_CO_2_ values were not different in patients with and without DCI. Current studies show that the pathogenesis of DCI is related to cortical spreading depolarizations and microcirculatory dysfunction [30]. These mechanisms are not closely linked to systemic P_a_O_2_ and P_a_CO_2_ which might explain our results. Keeping in mind that our P_a_CO_2_ values were mainly in the normal range, a different study showed that the maximum P_a_CO_2_ values were significantly different comparing patients with and without DCI [19]. We do not have data on microcirculation in our study, consequently the occurrence of a reversed Robin-Hood-phenomenon in SAH patients currently remains a hypothesis, which needs to be confirmed in future trials.

Higher ICP and lower cerebral perfusion pressure are another possible explanation for the worse outcome in patients with high-normal P_a_CO_2_ values. In our study, ICP values were not elevated in patients with higher P_a_CO_2._ Consequently, we do not believe that the detrimental effects of high-normal P_a_CO_2_ are mediated by ICP in our patient cohort, keeping in mind that ICP increases might have been buffered by additional drainage over a ventricular drain. Nevertheless, as reported before, patients with unfavorable outcome had higher ICP values compared to patients with favorable outcome in our study.

Detrimental effects through elevated P_a_CO_2_ levels might also have been mitigated by acidotic metabolism [18, 31, 32]. However, pH values were not significantly different in our study comparing patients with a median P_a_CO_2_ > 40 mmHg compared to ≤40 mmHg. While this shows that acidosis was not the mechanism causing the higher rate of unfavorable outcome it also leads to the question whether patients might have been adapted to higher P_a_CO_2_ values. We believe that there are two reasons why the pH values did not differ between patients with a P_a_CO_2_ > 40 mmHg compared to ≤40 mmHg. Firstly, our P_a_CO_2_ values were mainly within the normal range and therefore unlikely to change the pH considerably. Secondly, the base excess was significantly different between these groups, pointing out the fact that the mild changes in P_a_CO_2_ might have been buffered metabolically.

Regarding patients with aSAH who were mechanically ventilated, our cohort is representative compared to previously published cohorts. As shown in other studies [3, 33–35], known risk factors for outcome as higher values on the Hunt-Hess scale, longer ventilation time, occurrence of pneumonia and delayed cerebral ischemia were significant predictors of unfavorable outcome. Diameter of ruptured aneurysms in our study was comparable to other published data [3].

The most important limitation of our study is the single-center retrospective design, which restricts extrapolation of our results to other populations. The retrospective design and the generalized approach do not allow attribution of clear causality of our findings and the results are prone to selection bias. The results therefore are hypothesis-generating only. A selection bias has been introduced by including ventilated patients only. The rate of patients with a mRS scale score of 5 and 6 is higher compared to other studies [19, 36]. Only 11.3% of the patients had a favorable outcome while other publications could demonstrate a good clinical outcome with mRS < 3 in 59.5% [3]. This can be explained by the distribution of the Hunt-Hess score and the length of stay in our study. The percentage of patients with a Hunt-Hess score of 5 was 34% in our study vs. 10 and 11% in other published studies [19, 36]. The occurrence of DCI also seems higher in our population. However, it is expected that 74% of patients with a Hunt-Hess score ≥ 3 have DCI [37]. In our population this would lead to at least 89 expected cases (60%). Keeping in mind that these expected cases would include patients with unexpected neurological deterioration and not merely infarcts on CT, the rate of DCI is comparable to previously published studies. Although this explains the differences in outcome and in the occurrence of DCI it also limits the generalizability of our results. On the other hand, our study contains a high percentage of severely affected patients who are almost always ventilated and might benefit most from optimal P_a_CO_2_ levels. Even though we present a fairly large number of patients, some subgroups are small, limiting statistical power. In addition, regression analysis could only be conducted with a limited number of independent variables. Hence, not all imbalances shown in the univariate analysis could be adjusted for. Even the presented analysis is prone to model overfitting as we included 4 independent variables and the analysis must therefore be interpreted accordingly. Even though most of the P_a_CO_2_ values were within the normal range, some were higher than 45 mmHg or lower than 35 mmHg. We do not expect any relevant disturbances as it concerned only a minority of patients (9%).The degree of vasospasm was not recorded in our study which might have provided more information on the effect of vasospasm on clinical outcome. Another limitation is the irregular sampling of P_a_CO_2_ values during clinical routine. With a sampling frequency of roughly every two hours, it is possible that periods with increased or decreased P_a_CO_2_ values were missed. Using a dichotomous endpoint might have led to oversimplification. Even though scoring of the mRS was done blinded to this analysis, the physicians were not blinded to the clinical course.

## Conclusions

A higher proportion of P_a_CO_2_ values within the high-normal range (40–45 mmHg) was an independent negative predictor of favorable outcome in patients with aSAH who were mechanically ventilated in our study. This is an important novel finding as currently the whole normal range of P_a_CO_2_ values (35–45 mmHg) is considered to be beneficial. However, the results are hypothesis-generating only and need to be confirmed in a prospective trial to study the effect of P_a_CO_2_ in patients with SAH. If the results are reproduced, these findings will have the potential to change management of all ventilated aSAH patients. Future trials should also focus on effects of P_a_CO_2_ values on the microcirculation of the brain.

## Supplementary information

**Additional file 1: Table S1.** Comparison of P_a_CO_2_ and P_a_O_2_ by delayed cerebral ischemia (DCI; *n* = 150).

## Data Availability

The datasets generated and analyzed during the current study are available from the corresponding author on reasonable request.

## Abbreviations

ASA: Acetylsalicylic acid
aSAH: Aneurysmal subarachnoid hemorrhage
AUROC: Area under the Receiver Operating Characteristic curve
CBF: Cerebral blood flow
CI: Confidence interval
CT: Computed tomography
DCI: Delayed cerebral ischemia
EVD: External ventricular drain
F_i_O_2_: Fraction of inspired oxygen
ICP: Intracranial pressure
ICU: Intensive care unit
mRS: modified Rankin Scale
OR: Odds ratio
P_a_CO_2_: Arterial partial pressure of carbon dioxide
P_a_O_2_: Arterial partial pressure of oxygen
