# Supplementary material for: High-normal PaCO2 values might be associated with worse outcome in patients with subarachnoid hemorrhage – a retrospective cohort study
Source: BMC Neurol. 2020 Jan 20;20:31. doi: 10.1186/s12883-020-1603-0 (PMC6972024; doi:10.1186/s12883-020-1603-0)
Supplement: Supplementary file 1 — Additional file 1: Table S1. Comparison of PaCO2 and PaO2 by delayed cerebral ischemia (DCI; n = 150). [file 12883_2020_1603_MOESM1_ESM.docx]

Table S1 Comparison of PaCO_2_ and PaO_2_ by delayed cerebral ischemia (DCI; n=150)

|  | DCI (n=74) | no DCI (n=76) | *P^*^* |
| --- | --- | --- | --- |
| PaCO_2_ (mm Hg; p25; p75) | 39.3 (37.95; 41.85) | 39 (37; 41) | 0.1949 |
| PaO_2_ (mm Hg; p25; p75) | 99.08 (95; 108) | 99.95 (94.03; 108.5) | *0.6710* |
| *^*^*Wilcoxon rank-sum (Mann-Whitney) test | | | |
